# Macro- and Micronutrient Intake in Children with Avoidant/Restrictive Food Intake Disorder

**DOI:** 10.3390/nu13020400

**Published:** 2021-01-27

**Authors:** Ricarda Schmidt, Andreas Hiemisch, Wieland Kiess, Kai von Klitzing, Franziska Schlensog-Schuster, Anja Hilbert

**Affiliations:** 1 Behavioral Medicine Research Unit, Integrated Research and Treatment Center Adiposity Diseases, Department of Psychosomatic Medicine and Psychotherapy, Leipzig University Medical Center, 04103 Leipzig, Germany; anja.hilbert@medizin.uni-leipzig.de; 2 LIFE Leipzig Research Center for Civilization Diseases, Leipzig University, 04103 Leipzig, Germany; andreas.hiemisch@medizin.uni-leipzig.de (A.H.); wieland.kiess@medizin.uni-leipzig.de (W.K.); 3 Center for Pediatric Research, Hospital for Children and Adolescents, Leipzig University Medical Center, 04103 Leipzig, Germany; 4 Department of Child and Adolescent Psychiatry, Psychotherapy and Psychosomatics, Leipzig University Medical Center, 04103 Leipzig, Germany; kai.klitzing@medizin.uni-leipzig.de (K.v.K.); franziska.schlensog-schuster@medizin.uni-leipzig.de (F.S.-S.)

**Keywords:** ARFID, eating disorder, energy intake, food diary, children, adolescents, macronutrients, micronutrients, vitamin

## Abstract

Although case studies in avoidant/restrictive food intake disorder (ARFID) indicate severe nutritional deficiencies in those with a highly limited amount or variety of food intake, systematic analyses on food intake in treatment-seeking children and adolescents with ARFID are lacking. Within this study, *n* = 20 patients with an interview-based diagnosis of ARFID (0–17 years) were included and compared to *n* = 20 healthy controls individually matched for age and sex. Children or parents completed three-day food diaries and a food list. Macronutrient, vitamin, and mineral supply was determined based on the percentage of their recommended intake. The results showed a significantly lower total energy and protein intake in ARFID versus controls, with trends for lower fat and carbohydrate intake. ARFID subtypes of limited amount versus variety of food intake significantly differed in macro-, but not micronutrient intake. Those with ARFID met only 20–30% of the recommended intake for most vitamins and minerals, with significantly lower intake relative to controls for vitamin B1, B2, C, K, zinc, iron, and potassium. Variety of food intake was significantly reduced in ARFID versus controls in all food groups except carbohydrates. This study demonstrated that ARFID goes along with reduced everyday life macro- and micronutrient intake, which may increase the risk for developmental and health problems. Future studies additionally assessing serum nutrient levels in a larger sample may further explore differences in food intake across diverse ARFID presentations.

## 1. Introduction

With the release of the fifth edition of the Diagnostic and Statistical Manual of Mental Disorders (DSM-5) in 2013, avoidant/restrictive food intake disorder (ARFID) has been introduced in the newly combined feeding and eating disorder section [1]. ARFID extended and replaced the DSM-IV diagnosis of Feeding Disorder of Infancy and Early Childhood [2], now describing individuals across ages who show a persistently low amount and/or variety of food intake that may go along with other physical or mental impairments than weight loss only, including nutritional deficiencies, the need for supplemental feeding, and reduced psychosocial functioning. Notably, the avoidant or restrictive food intake cannot be accounted for by body image disturbances or weight loss intentions, and is not entirely attributable to a medical or mental disorder, a lack of food, or culturally accepted reasons such as a vegan diet. Recent research indicated a high prevalence of ARFID in samples seeking treatment for feeding or eating disorders and consistently revealed a range of mental comorbidities, including anxiety and neurodevelopmental disorders [3,4].

In addition, ARFID presents alongside a range of medical conditions. Several studies reporting on treatment-seeking individuals with ARFID clearly demonstrated nutritional deficiencies and related complications, such as dehydration and severe malnutrition due to a highly limited range of eaten foods [5,6,7] or a highly limited amount of oral food intake [8,9,10]. In a clinical sample of adolescents hospitalized for nutritional deficiencies, a significantly greater number of patients with ARFID were in need of enteral feeding compared with patients with anorexia nervosa [11], another highly restrictive eating disorder [1]. Blood samples of treatment-seeking individuals with ARFID were characterized by hypoproteinemia, hypophosphatemia, and hypokalemia, as well as vitamin D, B2, B12, A, C, E, K, iron, and folate deficiency, requiring them as vitamin or mineral replacement [5,6,8,11,12]. However, the nutritional status in treatment-seeking individuals with ARFID has neither been systematically nor consistently analyzed.

In fact, only two studies examined food intake in non-treatment-seeking individuals with ARFID based on a nutritional perspective. In a recent study, Harshman et al. analyzed data of four-day food records in a sample of 52 non-treatment seeking individuals aged 9 to 22 years (mean age 14 years) with full- or subthreshold interview-based diagnosis of ARFID, mostly within the normal weight range [13]. As a subthreshold diagnosis of ARFID was described as meeting all criteria for ARFID except criteria A1–A4 (i.e., weight loss/reduced growth, nutritional deficiency, supplemental feeding, psychosocial impairment), avoidant or restrictive food intake was not necessarily associated with significant health impairment. Although the amount of food intake in those with ARFID was not different from that of a control group without an eating disorder, dietary quality was characterized by a higher intake of foods high in carbohydrates and/or fat, including bread and sweets, and a lower intake of foods high in protein, including vegetables, compared with controls. Regarding micronutrient intake, individuals with full- or subthreshold ARFID had an absolute lower intake of vitamin K and B12 than controls, and a greater percentage of those with full- or subthreshold ARFID did not meet the national recommendations for magnesium and zinc intake. These data indicate that the sample by Harshman et al. was predominately comprised by individuals showing inadequate variety of food intake [13]. Especially selective eating behaviors may go along with specific nutritional deficiencies, while total energy intake may be adequate and effects on weight can be absent [14]. In a study by Schmidt et al. examining non-treatment seeking children between 8 and 13 years, those with interview-based, full-syndrome diagnosis of ARFID (*n* = 7) showed a considerably lower amount and variety of food intake than controls of the same age (*n* = 31) based on a recall of a representative day of eating for the past 28 days assessed via interview [15]. Unlike child report, showing significant group differences in recommended energy as well as macro- and micronutrient intake, parent-reported food intake identified significantly lower total energy intake, protein, fat, and zinc intake in those with ARFID versus controls, but no group differences for carbohydrates and other minerals. In contrast to the sample by Harshman et al., the majority of children by Schmidt et al. were underweight and were classified as limiting both amount and variety of food intake [13,15].

The little available evidence on nutritional and/or energy intake in ARFID relatively clearly indicated a reduced intake of protein compared with the recommendations and control groups and absent differences for carbohydrate intake, while the findings on total energy and fat intake were inconclusive, or may depend on whether the amount and/or variety of food intake are being limited. Serum levels in treatment-seeking individuals with ARFID and food intake data in non-clinical samples with ARFID further suggest deficiencies in vitamin intake, particularly vitamin D, B12, and K; as well as vitamin A, B2, C, and E; and mineral intake, including folate, zinc, phosphate, potassium, iron, and magnesium. In this context, the aim of this study was to quantify the amount and variety of food intake in treatment-seeking children and adolescents (0–17 years) with an interview-based diagnosis of full-threshold ARFID based on a three-day food diary and food list. It was hypothesized that children and adolescents with ARFID would consume a lower amount and variety of food than individually matched controls without an eating disorder. Specifically, it was expected that children would show a lower total energy and lower protein intake as well as a lower vitamin and mineral intake than controls, while fat and carbohydrate intake would not differ. Regarding food variety, it was hypothesized that children with ARFID would consume significantly less foods high in protein, including vegetables, fruits, and meat and fish, but more foods high in carbohydrates as well as processed foods, including sweets and snacks, and bakery products. Exploratorily, it was analyzed whether children’s nutritional and energy intake was different for the type of food restriction (amount or variety of food intake) in those with ARFID.

## 2. Materials and Methods

### 2.1. Participants

The study presents data on participants with a current ARFID diagnosis who were part of a validation study of the ARFID module for the Eating Disorder Examination [15]. Inclusion criteria for the main study were child age between 0 and 17 years, the presence (ARFID group) or absence (control group) of restrictive eating behaviors determined via telephone screening, and participation in a diagnostic interview on eating disorders including ARFID. The sample was recruited at the feeding and eating disorders unit of Leipzig University Medical Center, from a support group for individuals with ARFID and selective eating, and the population via adverts on the Internet or kindergarten, as well as the LIFE Child study [16]. To be included in the present study, children and adolescents were required to have a current diagnosis of ARFID determined via interview and having provided information on food intake via food diary. A total of 109 children and adolescents were enrolled in the treatment-seeking context between June 2018 and February 2020. Of them, 28 (26%) refused to participate for personal reasons (*n* = 13) or could not be contacted (*n* = 15). The remaining 81 children and adolescents and their parents were assessed via interview, revealing a current diagnosis of ARFID in 20 children and adolescents. All other participants received a lifetime diagnosis of ARFID, another eating disorder diagnosis (e.g., anorexia nervosa), or no diagnosis at all. Of *n* = 63 eligible children and adolescents from the community without restrictive eating behaviors, 20 were individually matched for age and sex to the ARFID group. All children ≥ 8 years and their parents provided written informed assent and consent prior to participation. The study was approved by the Ethics Committee of Leipzig University Medical Center (no. 120-15/ek).

ARFID diagnosis was based on the ARFID module of the Eating Disorder Examination (EDE), a standardized semi-structured interview with high reliability and validity [15]. Interviews were made with all parents and their children if > 7 years. As a main criterion for ARFID, the EDE-ARFID module evaluates a representative day of eating for each of the last three months, in addition to items on food variety and further diagnostic characteristics. Based on the ARFID module of the EDE, children can be classified as restricting the amount, variety, or both amount and variety of food intake. In addition to the ARFID module, the EDE in its adult [17,18] and child versions was administered [19,20] in order to evaluate the presence of other eating disorders than ARFID. All interviews were conducted by trained research assistants. Both parent and child reports were used to make a diagnosis of ARFID.

The ARFID group (*n* = 20) was 7.5 ± 5.3 years old, with 45% (*n* = 9) being female. Regarding objectively measured weight status, 45% (*n* = 9) were severely underweight, 15% were underweight (*n* = 3), and 40% had a normal weight (*n* = 8), with a mean standardized body mass index (BMI, kg/m^2^) of −1.5 ± 1.0 according to German age- and sex-specific reference data [21]. All children and adolescents with ARFID were of German nationality and were seeking treatment for eating or feeding disorders, with 6 (30%) or 14 (70%) of them receiving inpatient or outpatient treatment at the time of the study, respectively. A total of *n* = 6 (30%) children received supplemental feeding via tube or oral nutritional supplements; half of them received inpatient treatment. Regarding representativeness, the present sample was comparable to international, treatment-seeking samples of ARFID in terms of weight status, the proportion of boys and girls, and ethnicity (e.g., [4,22,23]).

Among those with ARFID, *n* = 11 (65%) were characterized by a limited variety of food intake, while *n* = 9 (45%) had a diet with a limited amount of food intake with or without limited variety. Both subtypes did not significantly differ in age, *t*(18) = −0.72, *p* = 0.482, or sex, Fisher’s exact test *p* = 0.653, although children with limited variety of food intake were descriptively older (8.3 ± 4.3 years versus 6.6 ± 6.4 years) and more likely to be male (64% versus 44%). Both ARFID subtypes were within the underweight range (−1.5 ± 1.0 versus −1.4 ± 1.0), without significant differences in mean standardized BMI, *t*(18) = −0.33, *p* = 0.746.

The control group was 7.5 ± 5.3 years old as well, included 45% (*n* = 9) girls, and had majorly normal weight (*n* = 13, 65%) with a mean standardized BMI of 0.0 ± 1.1. The ARFID and control group did not differ significantly in age, *t*(38) = 0.000, *p* = 0.999, or sex, Fisher’s exact test *p* = 0.999, but in mean standardized BMI, *t*(38) = −4.28, *p* < 0.001.

### 2.2. Measures on Food Intake

All participants were precisely instructed to prospectively complete a three-day food diary to document the amount of food intake prior to the diagnostic session. The days with a food diary were predetermined by the study team and included two weekdays and one weekend day in a non-consecutive order to increase representativeness of food intake. For children ≤ 13 years, parents filled in the food diary, while older adolescents completed the food diary by themselves. At the beginning of the diagnostic session, food diaries were inspected by a team member together with participants, in order to clarify unspecific information on portion size or other nutritional aspects.

Nutritional intake was calculated using PRODI^®^ v6 (Nutri-Science GmbH, Hausach, Germany), which is based on the German Nutrient Database (BLS, v3.02, Federal Research Center for Nutrition and Food, Karlsruhe, Germany) and includes energy and nutrient data on more than 15,000 foods. A researcher highly familiar with food diaries in eating and weight disorders matched the food and drink items documented in the food diaries to the database codes and portion sizes. Supplemental energy intake via tube feeding or oral nutritional supplements was not considered in analyses of food intake, in order to analyze children’s self-reliant oral food intake only. Because of the large age range, raw data on food intake were only reported for total energy intake in kcal for descriptive purposes, but were otherwise reported as the achieved proportion (%) of the recommended intake based on age- and sex-specific reference values for macro- and micronutrient intake [24]. Average values across all three days with a food diary were reported.

In addition, parents (for children ≤ 13 years) and adolescents were asked to report all the foods their child/they would currently eat based on a food list (unpublished, Sarah Eckhardt). The food list originally contained 14 food groups with a variety of foods listed. For this study, only seven major food groups were analyzed, excluding smaller food groups, for example, nuts, eggs, or soups. The number of eaten foods per food group was extracted with a maximum of 40 (meat, sausage, and fish), 38 (fruits), 36 (carbohydrates and bakery products), 34 (vegetables), 24 (sweets and snacks), and 14 (potato products and meals) possibly accepted foods per food group.

### 2.3. Statistical Analysis

Based on an assumed large effect for the amount and variety of food intake in ARFID versus controls previously reported in the literature [13,15], an intended test power of 1 − β = 0.80, α = 0.05, and an assumed correlation between groups of *r* = 0.30, *n* = 20 participants with ARFID had to be included [25].

All data were inspected for missing values and normal distribution. Data on the food list were lacking for *n* = 3 individuals with ARFID for organizational reasons, leaving *n* = 17 matched pairs for this analysis. Normality checks were carried out on residuals of the dependent variables, which were normally distributed for total energy intake and macronutrient data. Residuals for the majority of vitamins and minerals were positively skewed with a positive kurtosis.

Owing to individual matching, repeated measures analyses of variance (rmANOVA) were used to determine effects of group (within-subject factor: ARFID, controls) in food intake, specifically, in the percentage of total energy intake from protein, fat, and carbohydrates; the percentage of recommended intake of total kcal, protein, fat, and carbohydrates; and number of foods eaten per food group (dependent variables). For vitamin and mineral intake, Wilcoxon signed-rank tests were applied owing to dependent, but non-normally distributed data. Although ARFID may inherently go along with a low weight status, children’s standardized BMI was included as a covariate in sensitivity analyses due to significant group differences in the standardized BMI. Therefore, generalized linear models with an identity (energy and macronutrient intakes) or log link function (vitamin and mineral intake) for normal or gamma distribution with group as factor and standardized BMI as covariate were run. However, analyses with covariate will only be reported in the case of changing the main findings. In order to evaluate effects of ARFID subtype on food intake, Mann–Whitney *U* tests were conducted for total energy and macronutrient intake as well as vitamin and mineral intake with ARFID subtype (limited amount, limited variety) as the independent variable, without including any covariate based on relatively small group sizes.

An adjusted *p*-value was used for analyses on macronutrient data (four comparisons, *p* < 0.0125), vitamins (nine comparisons, *p* < 0.006), minerals (five comparisons, *p* < 0.01), and data on food variety (seven comparisons, *p* < 0.007) owing to multiple comparisons. For the evaluation of effect sizes, Cohen’s *d* and η_p_^2^ were used, indicating small (0.10 and 0.01), medium (0.50 and 0.06), and large effects (0.80 and 0.14) [26].

## 3. Results

### 3.1. Amount of Food Intake

Children and adolescents with ARFID consumed on average 1089 ± 689 kcal (range 0 to 2090, median 1322), which was significantly lower compared with the control group consuming 1469 ± 389 kcal (range 782 to 2166, median 1522), *F*(1, 19) = 7.807, *p* = 0.012, η_p_^2^ = 0.29. Similarly, the achieved percentage of recommended energy intake was significantly lower in those with ARFID than controls (*p* = 0.006) with a large effect, as shown in Table 1. Children and adolescents with ARFID consumed a descriptively lower percentage of total kcal from protein (large effect) and carbohydrates (small effect) compared with controls, without reaching the adjusted significance threshold (*p* > 0.0125). No group difference was seen in relative energy intake from fat (*p* > 0.0125, small effect). The mean percentage of protein intake based on age- and sex-specific reference values was within the recommended range in those with ARFID, but the control group showed a significantly higher protein intake (*p* = 0.003, large effect). The percentage of carbohydrate and fat intake relative to recommendations in the ARFID group was non-significantly lower compared with the control group (*p* > 0.0125, medium to large effects).

Regarding vitamin intake, group differences emerged in vitamin B1, B2, C, and K, with significantly lower values in the ARFID versus control group (*p* < 0.006, medium to large effects), while no differences were observed in the intake of vitamin B6, B12, D, E, and folate (*p* ≥ 0.006). Analyses of children’s mineral intake revealed significant group differences in zinc, iron, and potassium intake, with lower values in ARFID versus controls (*p* < 0.01, medium to large effects), while no differential effects emerged for calcium and magnesium intake. Detailed data on vitamin and mineral intake can be found in Table 2.

Differential analyses in those with ARFID identified specific characteristics related to the dietary intake as a function of ARFID subtype. Children and adolescents characterized by reduced amount of food intake were found to have a significantly lower intake of total energy in kcal (591.75 ± 521.17 vs. 1495.97 ± 530.92, *U* = 8, *p* = 0.001) and % (34.29 ± 26.99 vs. 89.65 ± 33.02, *U* = 8, *p* = 0.001), and macronutrients in % (protein, fat, and carbohydrate, *U* = 9–14, *p* = 0.002–0.013) than those with reduced food variety only. No significant group differences were seen for levels of recommended vitamin and mineral intake (*p* ≥ 0.052).

### 3.2. Variety of Food Intake

**Figure 1:**
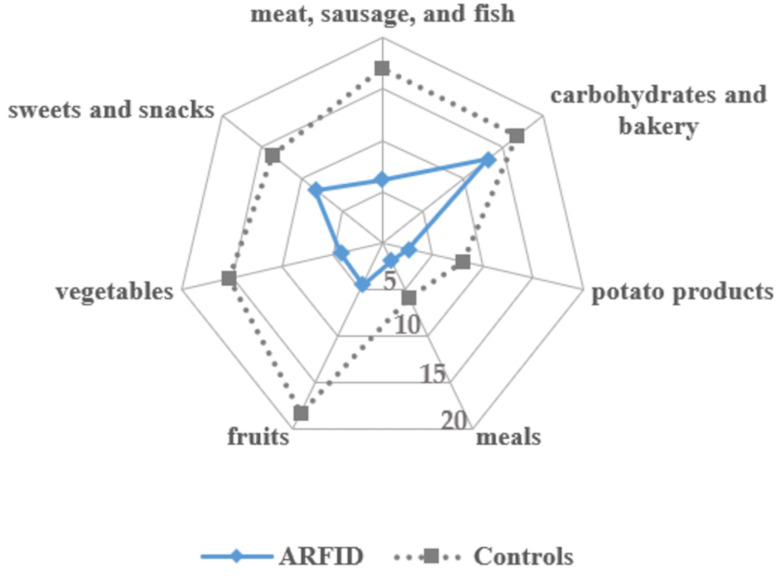
Variety of food intake in avoidant/restrictive food intake disorder (ARFID) and individually matched controls depicting the number of accepted foods per food group.

Among foods groups that were consumed most often by children and adolescents with ARFID were carbohydrates and bakery products as well as sweets and snacks, while meals and potato products were solely eaten. In the control group, fruits, meat, sausage, and fish, as well as carbohydrates and bakery products were the food groups with the highest number of accepted foods. As depicted in Figure 1, the ARFID group had a significantly lower range of accepted foods than the control group for meat, sausage, fish, potato products, vegetables, fruits, and meals, *F*(1, 14) = 10.346–52.972, *p* = 0.001–0.006, η_p_^2^ = 0.43–0.79. No significant group differences emerged for carbohydrates and bakery products, *F*(1, 14) = 2.987, *p* = 0.106, η_p_^2^ = 0.18.

## 4. Discussion

For the first time, this study analyzed nutritional and energy intake in a treatment-seeking sample of children and adolescents with a full-threshold diagnosis of ARFID compared with typically developing controls individually matched for age and sex. As hypothesized, data of a three-day food diary indicated that ARFID was associated with a significantly lower total energy intake, in terms of both kcal and the achieved percentage of the age- and sex-specific recommendations. The breakdown of ARFID’s total energy intake into macronutrients was not different from that of the control group and was largely in line with the recommended relative intake of macronutrients. However, children and adolescents with ARFID had a significantly lower intake of specific vitamins and minerals compared with controls. Regarding food variety, children with ARFID had an expectedly significantly lower range of accepted foods than controls for all food groups, except for carbohydrates and bakery products. The study thus provides valuable information on the quantity and quality of food intake in children and adolescents with ARFID, highlighting the severe impact that ARFID-related restrictive and avoidant eating behaviors may have on physical and mental health.

As a key defining feature of ARFID, the persistent failure to meet appropriate nutritional and/or energy needs was mirrored in children’s everyday life food intake. Specifically, treatment-seeking children and adolescents meeting criteria for ARFID met about 65% of the recommended energy intake based on age- and sex-specific reference values, compared with 99% achieved by the control group. Depicting the heterogeneity in ARFID, the range of energy intake through oral intake was very large in those with ARFID, varying from total enteral feeding to higher than recommended energy intake. In this context, we provided evidence that energy intake in those with a limited variety of food intake only was relatively acceptable (89%) and that those characterized by limited amount of food intake indeed consumed a low percentage of recommended energy intake (34%). These differences may have profound effects for the course of ARFID, in respect to both its severity and progression rate. Regarding macronutrient composition, there was a trend that children with ARFID received less calories from protein than controls, while the percentage of calories from fat did not differ between ARFID and controls, as previously found [13]. In contrast to Harshman et al. [13], however, in the present sample, percent calories from carbohydrates was not significantly higher in the ARFID group, which may be related to a lower relative proportion of those with a reduced variety of food intake. Children with ARFID consumed significantly less protein than controls, although achieving 105% of the recommended intake, which is line with the findings of Harshman et al. [13]. Notably, children and adolescents with ARFID only met 59% and 78% of recommended intake for carbohydrates and fat, respectively, which may mirror the inclusion of children and adolescents with ARFID based on limited amount of food intake. At the same time, these average values correspond to < 10th percentile in macronutrient intake compared with German reference data in 10- to 15-year-olds [27], although it should be noted that the ARFID group had a high variability in macronutrient intake.

In line with hypotheses, children and adolescents with ARFID had a lower range of accepted foods according to self- or parent-report, which was based on a food list that included 200 different foods from seven food groups. Rather than assessing the diversity of a diet based on evaluating a number of food diaries, the food list asked for currently accepted foods, and thus depicts a conceptually slightly different kind of food variety than that of Harshman et al. [13], who used a four-day food record to analyze dietary variety. Owing to seasonal variations in the availability and consumption of various foods (e.g., fresh fruits or ice cream during summertime, hearty food in the wintertime), the food list is especially able to portray a variety of foods that is independent of those variations. However, it is important to note that analyzing a large number of random food diaries is well suited for mapping specific dietary patterns including food variety. Consistent with the findings in a non-treatment-seeking sample with ARFID [13], this study found that foods high in carbohydrates, including sweets and snacks, processed foods, and bakery products, were accepted most among all food groups in those with ARFID. The number of accepted foods in these food groups did not differ from that of the control group. Other food groups were significantly less consumed compared with healthy controls, which may help explain nutritional deficiencies reported previously in clinical and case samples with ARFID [5,6,8,11,12] as well as the present results on vitamin and mineral intake. At the same time, the results on food variety provide theoretical support for the presence of ARFID in individuals with overweight or obesity, who may have a highly limited range of high-calorie foods with low micronutrient density.

As hypothesized, vitamin intake in ARFID was lower compared with individually matched controls, which is consistent with a range of case studies reporting on severe nutritional deficiencies based on serum levels in treatment-seeking children and adolescents with ARFID (e.g., [5,6,28]). Specifically, intake of vitamin B1, B2, C, and K was lower in ARFID versus controls. As vitamin B1, also known as thiamine, is relevant for glucose metabolism and neural functions, there is an increased need for vitamin B1 for individuals with a carbohydrate-rich nutrition, as may be the case with ARFID. At the same time, reduced intake of offal, legumes, and green vegetables as the main sources of vitamin B1 may contribute to vitamin B1 deficiency. Vitamin B2, also called riboflavin, plays a vital role in energy metabolism as well and can be most effectively absorbed through meat, dairy products, or leafy green vegetables. Group differences in vitamin B2 intake may thus result from a lower range of accepted meat and vegetables. Similarly, the limited variety of foods from vegetables and fruits in ARFID may be related to a lower vitamin C intake compared with controls, which may affect reduced immune function. In line with Harshman et al. [13], ARFID was associated with lower vitamin K levels, likely due to a lower intake of (green) vegetables in ARFID compared with controls. Notably, previous case studies in ARFID consistently highlighted a vitamin D deficiency; however, when compared with healthy controls, no group differences in nutrition-based vitamin D intake emerged, both in the present study and in Harshman et al. [13]. This may be due to the relatively high prevalence of vitamin D deficiency and insufficiency in the general pediatric population [27,29] or inappropriate reference values beyond the fact that food intake alone typically does not provide required amounts of vitamin D. Non-significantly different levels in folate, B6, or B12 (cobalamin) may be related to a generally high availability of, for example, B6 in a wide range of foods or a relatively high B12 body storage, which becomes critical especially in the case of long-term undersupply. Mineral intake in children and adolescents with ARFID was lower for zinc, iron, and potassium, mirroring recent findings from clinical and case studies [12,13] and the population [13,15], placing them at risk for impaired cognitive and physical performance and related development.

Despite several strengths of the study including the clinical sample with an interview-based diagnosis of ARFID, a three-day food record on predefined, non-consecutive days, and the individual matching design, some limitations have to be mentioned. Although the study was adequately powered for detecting large effects in a repeated measures design, a larger sample would allow for detecting medium-sized effects, which may have clinical implications as well. At the same time, a narrower age range would allow for absolute comparisons in nutrient intake; currently, absolute comparisons were deemed inadequate because of different nutritional needs in children and adolescents aged 0 to 17 years. As already noted, a three-day food diary was used, which is an internationally recommended method for estimating food intake (e.g., [29,30]), and produced highly reliable values in the control group relative to reference data from the population [27], the additional assessment of serum micronutrients would complement the data on food intake. In this context, medical comorbidities and medication intake with a likely effect on energy metabolism were not considered systematically in this study. Finally, as the study was cross-sectional in design, we were neither able to portray the course of the nutritional status over time, for example, over the course of treatment, nor to identify differences in food intake in those with and without remission from ARFID.

## 5. Conclusions

In sum, this study demonstrated that children and adolescents seeking treatment for ARFID were characterized by an inadequate amount and variety of food intake compared to individually matched healthy controls. Although the ARFID group was characterized by a lower energy intake from protein than controls, protein intake was within the recommended ranges. However, children and adolescents with ARFID showed a lower intake of the majority of vitamins and minerals compared with controls, which was further supported by a lower range of accepted foods for all food groups except carbohydrates and bakery products. While children and adolescents with ARFID with a reduced variety of food intake had a relatively acceptable total energy intake via oral intake, they showed a substantially lower vitamin and mineral intake than controls, comparable to that of children and adolescents with ARFID, who were described by a limited amount of food intake. Clinically, the results indicated that children and adolescents with ARFID may have an undersupply of specific vitamins and minerals, which may increase their risk for nutritional deficiencies and need for supplementation, especially in younger children and long-term undersupply. Although monitoring of children’s food intake is standard within a multidisciplinary inpatient treatment, clinicians should have a focus on their patients’ nutritional supply through oral intake during outpatient psychological treatment as well, ideally via both lab reports and food diaries. Compared with individuals with anorexia nervosa, children and adolescents with ARFID seem to consume more energy from fat, but less calories from protein [31,32,33], which should be examined in more detail in future studies. The quality and quantity of a patient’s diet may likely be a differential indicator for eating disorder diagnosis. Scientifically, future studies are welcomed to further examine nutritional and energy intake in individuals with ARFID using more objective methods than food records, such as the doubly labeled water method, and to gain more insight into the factors driving reduced food and energy intake. Based on recent evidence highlighting that biological abnormalities in sensory perception, homeostatic appetite, and fear responsiveness may underlie ARFID-related eating behaviors [34], it might be valuable to systematically explore differences in food intake across diverse ARFID presentations and to obtain information about specific dietary patterns in those with, for example, a lack of interest in eating or fear of aversive consequences. Eventually, a child’s diet may be predictive of specific ARFID presentations both cross-sectionally and longitudinally, or may predict treatment success.

## Figures and Tables

**Table 1:** Total energy consumed from protein, fat, and carbohydrates and achieved percentage of recommended energy and macronutrient intake in avoidant/restrictive food intake disorder (ARFID) and controls.

	Controls		ARFID		*F*(1, 19)	*p*	η_p_^2^
	*M* ± *SD*	Range	*M* ± *SD*	Range			
% of total energy intake ^1^							
Protein	12.3 ± 2.0	8.6–16.7	10.1 ± 3.6	0–16.4	5.352	0.033	0.23
Fat	34.1 ± 5.9	23.1–46.1	34.0 ± 14.3	0–68.8	0.001	0.972	0.00
Carbohydrates	52.1 ± 6.7	41.2–66.4	49.6 ± 16.8	0–75.9	0.388	0.541	0.02
% of recommended intake ^1^							
Total kcal	99.3 ± 24.5	47.2–150.4	64.7 ± 41.0	0–149.3	9.510	0.006	0.33
Protein	195.3 ± 84.8	80.8–324.3	104.8 ± 77.7	0–282.3	12.147	0.003	0.40
Fat	103.2 ± 31.9	62.5–174.0	78.1 ± 63.7	0–229.0	3.106	0.095	0.15
Carbohydrates	91.6 ± 28.8	34.5–172.1	58.9 ± 37.6	0–124.3	7.319	0.014	0.29

^1^ Reference values are based on German age- and sex-specific recommendations for energy intake.

**Table 2:** Achieved percentage of recommended vitamin and mineral intake in avoidant/restrictive food intake disorder (ARFID) and controls.

	Controls		ARFID		*Z*	*p*	|*d*|
	*M* ± *SD*	Range	*M* ± *SD*	Range			
% of vitamin intake ^1^							
B1	60.9 ± 52.4	14.6–238.5	19.4 ± 21.8	0–67.8	−3.114	0.002	1.0
B2	41.5 ± 23.8	13.0–96.9	18.7 ± 18.6	0–67.8	−3.245	0.001	1.1
B6	61.2 ± 48.0	11.1–199.7	24.3 ± 21.4	0–79.2	−2.504	0.012	1.0
B12	42.7 ± 31.9	3.8–123.0	29.1 ± 62.8	0–269.2	−2.330	0.020	0.3
C	143.6 ± 138.1	21.1–491.4	42.7 ± 36.1	0–133.4	−3.541	< 0.001	0.7
D	5.3 ± 8.6	0.5–38.6	8.0 ± 17.1	0–67.1	−0.762	0.446	0.2
E	33.6 ± 23.0	8.7–94.5	25.0 ± 28.8	0–101.2	−1.285	0.199	0.3
K	64.5 ± 45.0	13.5–152.6	23.8 ± 18.6	0–65.1	−2.896	0.004	1.2
Folate	39.7 ± 21.7	13.0–95.9	18.7 ± 27.0	0–118.7	−2.373	0.018	0.9
% of mineral intake ^1^							
Zinc	52.5 ± 28.7	11.4–122.6	21.1 ± 30.9	1.2–134.9	−3.070	0.002	1.1
Calcium	47.4 ± 32.2	13.1–116.6	51.0 ± 60.3	3.0–210.5	−0.174	0.862	0.1
Iron	30.6 ± 16.5	8.9–79.8	17.4 ± 22.8	0.2–102.2	−2.635	0.008	0.7
Magnesium	71.5 ± 38.8	22.1–154.9	68.8 ± 70.1	11.8–251.2	−0.283	0.777	0.0
Potassium	42.3 ± 24.3	13.1–116.6	20.3 ± 15.7	0.5–56.0	−2.678	0.007	2.0

^1^ Reference values are based on German age- and sex-specific recommendations for energy and nutrient intake.

## Data Availability

The data presented in this study are available on request from the corresponding author.
